# Utilizing magnetic xanthan gum nanocatalyst for the synthesis of acridindion derivatives via functionalized macrocycle Thiacalix[4]arene

**DOI:** 10.1038/s41598-023-49632-x

**Published:** 2023-12-13

**Authors:** Fereshte Hassanzadeh-Afruzi, Mohammad Mehdi Salehi, Ghazaleh Ranjbar, Farhad Esmailzadeh, Peyman Hanifehnejad, Mojtaba Azizi, Faten Eshrati yeganeh, Ali Maleki

**Affiliations:** Catalysts and Organic Synthesis Research Laboratory, Department of Chemistry, Iran University of Science and Technology, Tehran, Iran

**Keywords:** Chemistry, Materials science, Nanoscience and technology

## Abstract

An effective method for synthesizing acridinedione derivatives using a xanthan gum (XG), Thiacalix[4]arene (TC4A), and iron oxide nanoparticles (IONP) have been employed to construct a stable composition, which is named Thiacalix[4]arene-Xanthan Gum@ Iron Oxide Nanoparticles (TC4A-XG@IONP). The process used to fabricate this nanocatalyst includes the *in-situ* magnetization of XG, its amine modification by APTES to get NH_2_-XG@IONP hydrogel, the synthesis of TC4A, its functionalization with epichlorohydrine, and eventually its covalent attachment onto the NH_2_-XG@IONP hydrogel. The structure of the TC4A-XG@IONP was characterized by different analytical methods including Fourier-transform infrared spectroscopy, X-Ray diffraction analysis (XRD), Energy Dispersive X-Ray, Thermal Gravimetry analysis, Brunauer–Emmett–Teller, Field Emission Scanning Electron Microscope and Vibration Sample Magnetomete. With magnetic saturation of 9.10 emu g^−1^ and ~ 73% char yields, the TC4As-XG@IONP catalytic system demonstrated superparamagnetic property and high thermal stability. The magnetic properties of the TC4A-XG@IONP nanocatalyst system imparted by IONP enable it to be conveniently isolated from the reaction mixture by using an external magnet. In the XRD pattern of the TC4As-XG@IONP nanocatalyst, characteristic peaks were observed. This nanocatalyst is used as an eco-friendly, heterogeneous, and green magnetic catalyst in the synthesis of acridinedione derivatives through the one-pot pseudo-four component reaction of dimedone, various aromatic aldehydes, and ammonium acetate or aniline/substituted aniline. A combination of 10 mg of catalyst (TC4A-XG@IONP), 2 mmol of dimedone, and 1 mmol of aldehyde at 80 °C in a ethanol at 25 mL round bottom flask, the greatest output of acridinedione was 92% in 20 min.This can be attributed to using TC4A-XG@IONP catalyst with several merits as follows: high porosity (pore volume 0.038 cm^3^ g^−1^ and Pore size 9.309 nm), large surface area (17.306 m^2^ g^−1^), three dimensional structures, and many catalytic sites to active the reactants. Additionally, the presented catalyst could be reused at least four times (92–71%) with little activity loss, suggesting its excellent stability in this multicomponent reaction. Nanocatalysts based on natural biopolymers in combination with magnetic nanoparticles and macrocycles may open up new horizons for researchers in the field.

## Introduction

The N-containing heterocyclic structures have been considered essential members of organic materials. They are the main scaffold of a wide spectrum of medicine and biologically active products^1–3^. These compounds have been attracted great attention for several decades because of their extensive applications in numerous fields, including medicine, veterinary products, disinfectants, and antioxidants^4–7^. In past years, multicomponent reactions (MCRs) have attracted the curiosity of pharmaceutical scientists, particularly for the synthesis of heterocyclic compounds with significant variety, for instance, Hantzsch and acridinedione derivatives^8,9^. Among the many medicinal and biological properties of acridinediones, they possess a wide range of activities such as ionotropic activity promoting calcium entry into cells, anticancer activity, inhibition of enzymes and tumor cells, antimicrobial activity and cytotoxicity^10^. Several pharmaceuticals can be synthesized from 1,4-dihydropyridines (1,4-DHPs), often used in synthetic reactions as intermediates^11^. Different methods produce acridine derivatives (ADs), which are usually hazardous, expensive, and take quite a while to synthesize. The manufacturing of the diverse variety of chemicals that are essential to our contemporary society depends greatly on heterogeneous catalysts^12,13^. In addition to their selectivity, thermal stability, high surface area, high activity, nontoxicity, and recyclability for repeated reaction cycles, heterogeneous catalysts have a range of advantages as well^14^. The acridinedione moiety plays an instrumental role in synthesizing of bioactive heterocyclic molecules. Through designing and developing MCRs synthesis using sustainable principles and techniques and biobased catalysts, high-quality synthesis can be achieved. Ethyl acetoacetate, hydrazine hydrate, aldehydes, and malononitrile have been used in the MCRs process to synthesize ADs. g-C_3_N_4_@L-arginine^15^, Fe_3_O_4_@Polyaniline-SO_3_H^16^, GO/CR-Fe_3_O_4_^17^, ionic liquids^18^, The aforementioned process began with catalysts such as imidazole^19^ and tetranitrophenol. The fact that bio-based catalysis is environmentally benign and has excellent biocompatibility, and chemical diversity over conventional catalysis, as a result of its benefits, including high activity and selectivity. The development of natural polymer-based catalysts has received attention recently due to their remarkable advantages such as their eco friendliness, hydrophilicity, abundance, availability, and low cost. Previous research has shown, a polysaccharide including Arabic Gum^20^, sodium alginate^21^, cellulose^22^, dextrin^23^, and chitosan^24^; their modified forms were commnoly employed as main components of the different catalytic systems^25–27^. Additionally, a wide range of hydratererocylic compounds can be synthesized via catalysis using magneticnatural polymer composites, including magnetic guanidinylated chitosan (MGCS) nanobiocomposite^28^, HNTs/Chit catalyst^29^, Hyd AG-g-PAN/ZnFe_2_O_4_^30^, and Fe_3_O_4_@xanthan nanocatalyst^31^.

Xanthan gum (XG) a prominent example of polysaccharide that has attracted much attention in various industries and fields, including cosmetics, tissue engineering, drug delivery, wound healing, and the food industry^32^. Despite its incredible advantages, XG has several limitations, such as low specific surface area, heat resistance, difficulty in processability, insolubility in common organic solvents, instability and relatively poor hydrodynamic volume^33^. The chemical functionalization of this natural polymer by magnetic nanomaterials, supramolecules, synthetic polymers and also network formation of the natural polymers can be effectively enhancing their structural and chemical properties making them a promising candidate for organic reaction^34,35^.

Thiacalix[4]arenes (TC4A) are three-dimensional basket-like compounds considered a new category of calixarenes, a third generation of supramolecules. These are tetramers of phenols linked by sulfur atoms and have been identified as a prominent ligand for the construction of coordination cages and molecular clusters^36^. Indeed, the inclusion of sulfur atoms instead of the methylene bridges makes thiacalix[4]arenes particularly intriguing compounds, with with remarkable structural features that differ from the chemistry of "traditional" calixarenes ^37^. The TC4As are fascinating compounds with changeable conformations and excellent coordination ability, cavity structure, host–guest properties, versatile derivatization and high heat resistance has several applications in supramolecular chemistry^36,38^. The combination of TC4A with other materials and compounds can be used to construct different composites as sensors, absorbers, and catalysts in organic reactions^39,40^. Several catalytic systems based on calixarene have been shown to have effective catalytic characteristics for various organic processes.

**Figure 1:**
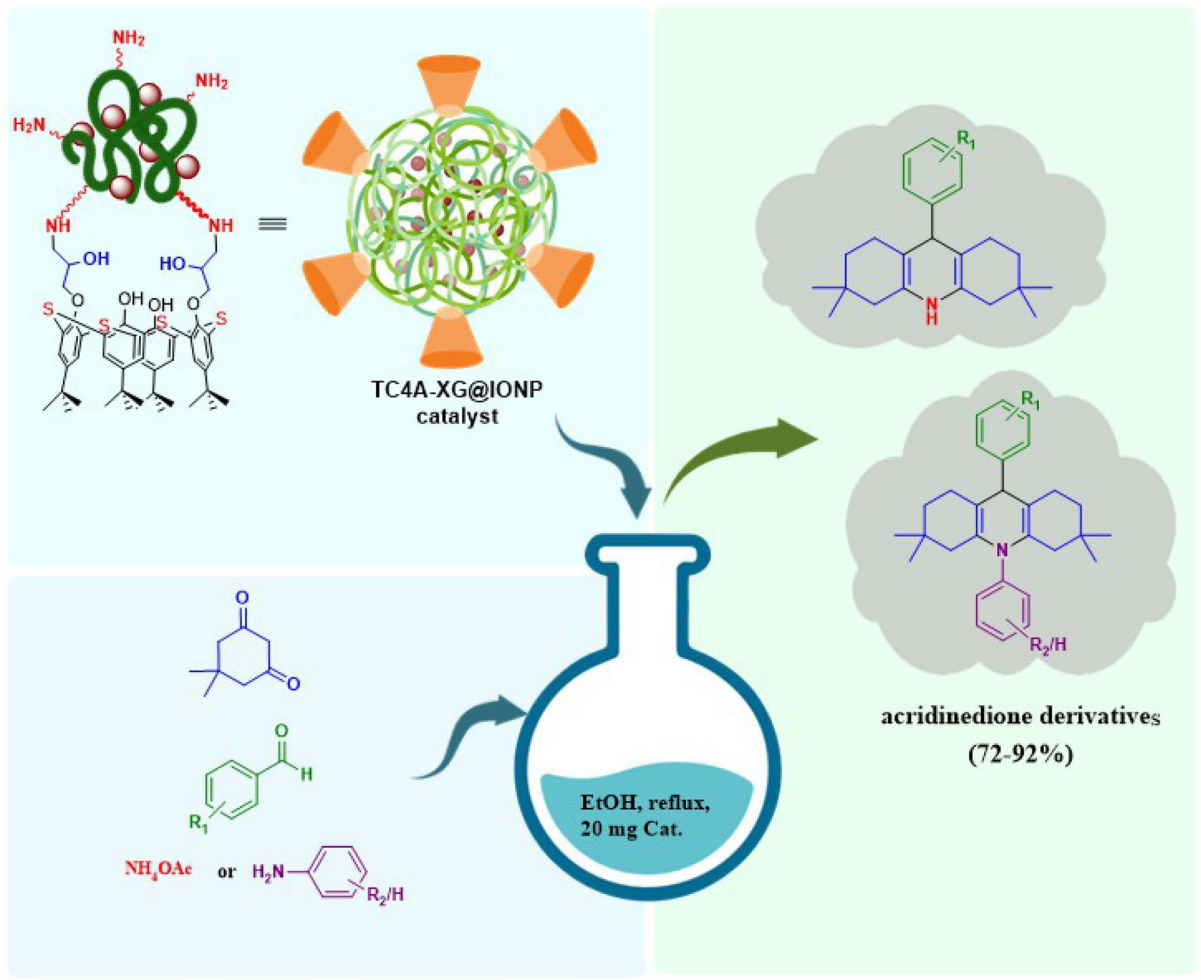
The graphical abstract of Utilizing Magnetic Xanthan Gum Nanocatalyst for the Synthesis of Acridindion Derivatives via Functionalized Macrocycle Thiacalix[4]arene.

Hence, in continuing our efforts in designing and preparing the natural-polymer based heterogeneous catalyst, we aimed to construct a composed of the amine-modified XG, iron oxide nanoparticles, and TC4A, based on the covalent attachments using epichlorohydrin as organic linkers as illustrated in Fig. 1. The process used to fabricate the TC4A-XG@IONP Nanocatalyst including: (a) *in-situ* magnetization of XG, (b) its amine-modification by APTES, (c) the synthesis of TC4A, (d) its functionalization with ECH as a crosslinking agent, and eventually its covalent attachment onto the NH_2_-XG@IONP hydrogel matrix. The structure and physicochemical characteristics of the fabricated catalyst have been characterized by utilizing different analyses. The catalytic activity of the designed nanocatalyst have been assessed for the acridinedione derivatives. In this pseudo-fourcomponent condensation, a combination of ethyl acetoacetate, various aromatic aldehydes, ammonium acetate/aniline or substituted aniline, and NH_4_OAc were reacted to the synthesis acridinedione derivatives. It has been observed that all are synthesized with good to excellent yields by utilizing the designed TC4A-XG@IONP nanocatalyst over a short reaction times.

## Experimental

### Materials and instruments

Xanthan gum (XG) (Sigma-Aldrich, Sigma-Aldrich Cas.no (G1253)), Ferric chloride hexahydrate (FeCl_3_·6H_2_O) (ACS reagent, 97%), (3-Aminopropyl) triethoxysilane (APTES) (≥ 98%, Sigma-Aldrich),Tetraethylene glycol dimethyl ether (≥ 99%, Sigma-Aldrich), Para-tert-butyl-phenol (99%, Sigma-Aldrich), Sodium hydroxide (reagent grade, ≥ 98%, pellets (anhydrous), Sigma-Aldrich), Sulfur (powder, 99.98% trace metals basis), Toluene (anhydrous, 99.8%, Sigma-Aldrich), Ethanol (96%, Sigma-Aldrich), Sulfuric acid (ACS reagent, 95.0–98.0%, Sigma-Aldrich), Diethyl ether (ACS reagent, anhydrous, ≥ 99.0%, contains BHT as an inhibitor, Sigma-Aldrich), Ammonia (puriss., anhydrous, ≥ 99.9%,Sigma-Aldrich), Epichlorohydrin (ECH) (purum, ≥ 99% (GC), Sigma-Aldrich).

FT-IR spectroscopy (Shimadzu FT-IR-8400S), EDX spectroscopy(Numerix DXP–X10P), VSM analysis (Meghnatis Kavir Kashan Co), BET analysis (BELCAT-A), XRD (DRON-8 X-ray diffractometer), EDX (VEGA-TESCAN-XMU), FESEM (Hitachi S-5200 and ZELSS SIGMA), TGA (BAHR-STA 504), Oven (Genlab Ltd), Zeta Meter (Zeta Meter Inc), H-NMR (VARIAN,INOVA 500 MHz).

### Catalyst preparation

#### Preparation of IONP@XG

For preparing magnetic XG by co-precipitation method, 0.4 g XG was added to 85 ml d.w. in a double-mouthed flask. The temperature was raised to 45 °C until it was completely dissolved. FeCl_2_.4H_2_O (0.396 g, 2 mmol), FeCl_3_.6H_2_O (0.54 g, 4 mmol), and 10 ml d.w. were added to the mixture and stirred for 30 min to dissolve. Then, it was placed under a N_2_ atmosphere, and the temperature was raised and fixed at 80 °C. The mixture was stirred for 1 h after adding 8 ml of ammonia dropwise. After that, it was washed 10 times with d.w. and ethanol after that dried in an oven over 65 °C. Finally, a brown powder (IONP@XG) was obtained.

#### ***Preparation of IONP@XG-NH***_***2***_

0.5 g IONP@XG, 2 ml APTES, and 20 ml ethanol were poured into a round bottom flask and refluxed for 48 h. Then, it was washed seven times with ethanol, dried in an oven, and a brown powder (IONP@XG-NH_2_) was obtained.

#### Preparation of TC4A-XG@IONP

**Figure 2:**
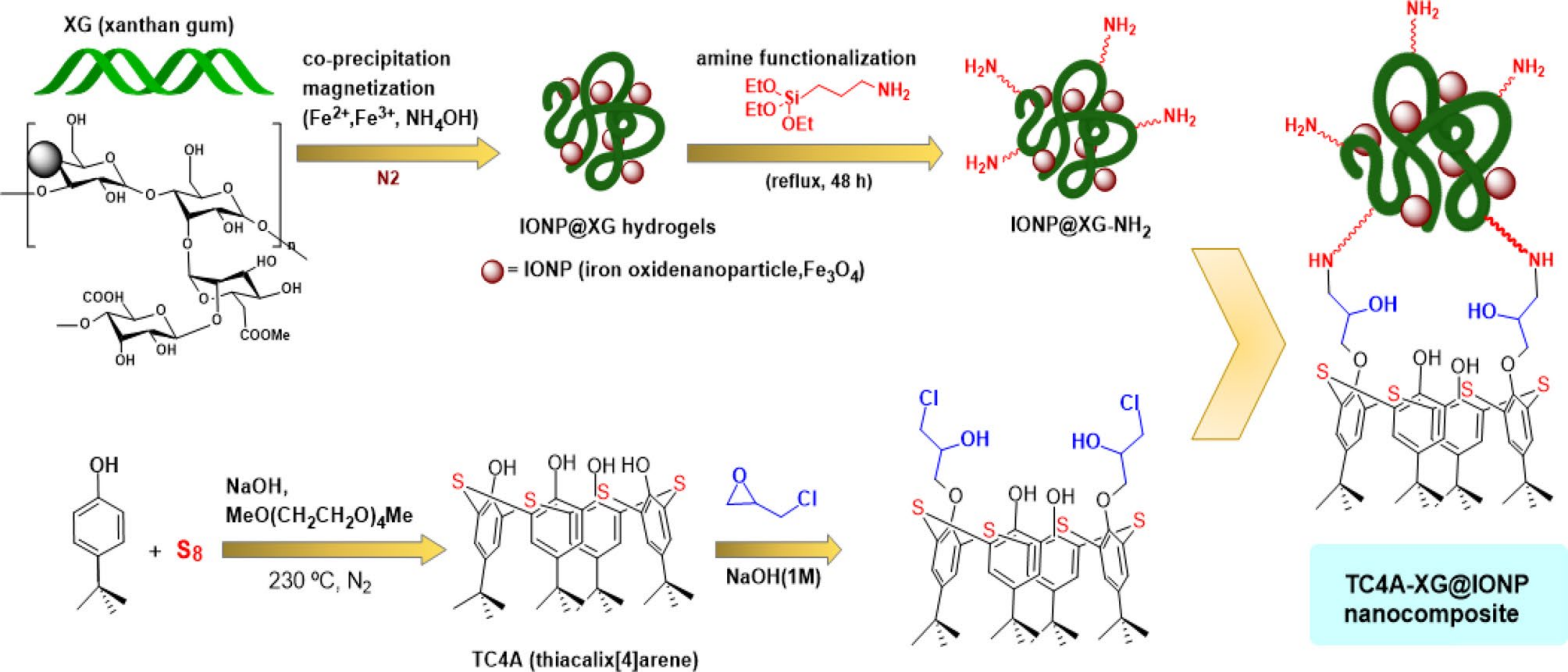
Schematic pathway for the fabrication of the TC4A-XG@IONP nanocatalyst.

0.36 g prepared thiacalix[4]arene (TC4A was synthesized based on the reported procedure in literature^41^) and 35 ml ethanol were added to a 250 ml round bottom flask with a solution of NaOH (1 M) (to make the mixture basic) and stirred for 20 min. Next, 0.1 ml epichlorohydrin was added to the mixture, and litmus paper was used to check the basic of the mix. Then, the temperature was raised to 60 °C, and the mixture was stirred for 3 h. After that, 0.5 g IONP@XG-NH_2_ and 25 ml distilled water were added and stirred for 16 h. Then, the mixture was filtered, washed with distilled water and ethanol, and dried in an oven. Finally, a dark brown powder (TC4A-XG@IONP) was obtained. The preparation route of the TC4A-XG@IONP nanocatalyst is illustrated in Fig. 2.

#### General procedure for the synthesis of acridinedione derivatives

A 25 ml round bottom flask was filled with a combination of aldehyde (1 mmol), dimedone (2 mmol), ammonium acetate/aniline/substituted aniline (1 mmol), TC4A-XG@IONP catalyst (0.01 g), and ethanol (5 ml), and the liquid was refluxed at 80 °C for 20 min. TLC kept track of the reaction's development. The combination was progressively cooled to room temperature when the reaction was finished. The nanocatalyst was then removed using external magnetic force, and the desired product was produced by recrystallizing with ethanol.

### Selected spectral data

#### 9-(4-chlorophenyl)-3,3,6,6-tetramethyl-10-(o-tolyl)-3,4,6,7,9,10-hexahydroacridine-1,8(2H,5H)-dione (6 g)

**FT-IR (KBr, cm**^−**1**^**):** 2952, 2870, 1635, 1579, 1489, 1222, 1087, 837, 737 cm^−1^. ^**1**^**H NMR (500 MHz, DMSO): δ** H (ppm) = 0.74(s, 6H, 2 CH_3_), 0.86(s, 6H, 2 CH_3_), 1.07(s, 3H, CH_3_), 1. 50(d, 2H, 2 CH), 2.21(m, 6H, 6 CH), 5.05(s, 1H, CH), 6.96–7.50(m, 8H, Ar–H) (Figs. S1 and S2).

#### 3,3,6,6-Tetramethyl-9-(4-chlorophenyl)-3,4,6,7,9,10-hexahydroacridine1,8(2H,5H)-dione (5c)

**FT-IR (KBr, cm**^−**1**^**):** 2875, 2801, 1606, 1490, 1221, 1066, 887, 763 cm^−1^. ^**1**^**H NMR (500 MHz, DMSO): δ** H (ppm) = 0.68(s, 6H, 2 CH_3_), 0.85(s, 6H, 2 CH_3_), 1. 73(d, 2H, 2 CH), 1,98(d, 2H, 2 CH), 2.16(d, 2H, 2 CH), 2.21(d, 2H, 2 CH), 5.02(s, 1H, CH), 7.15–7.35(m, 4H, Ar–H), 7.42(b, 2H, Ar–H), 7.55–7.64(m, 3H, Ar–H) (Figs. S3 and S4).

## Result and discussion

### Characterization

#### Fourier-transform infrared spectroscopy analysis

**Figure 3:**
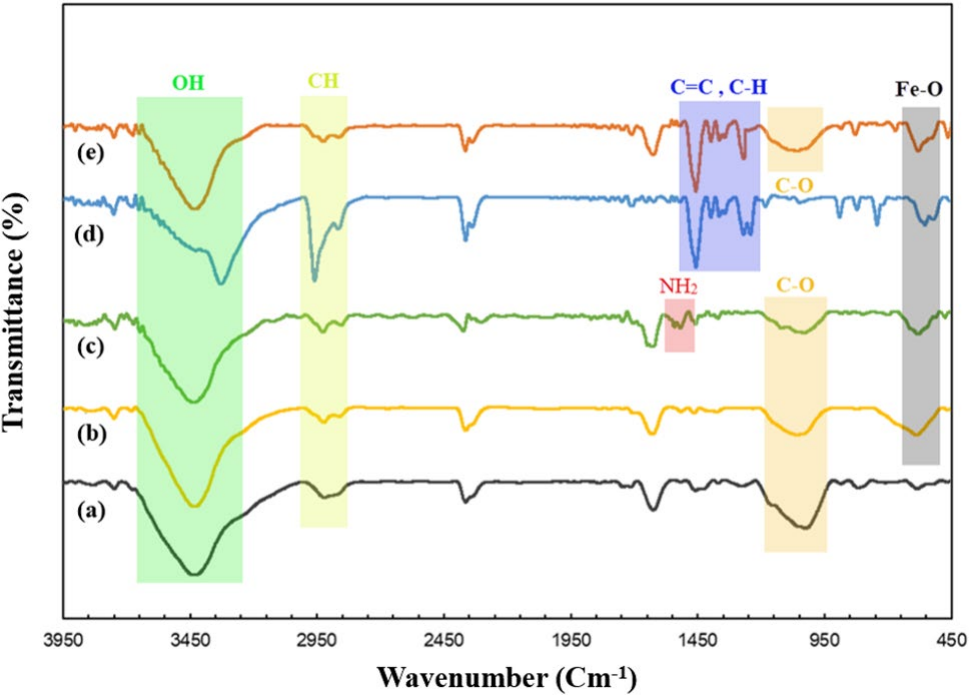
The FT-IR spectra of (**a**) XG, (**b**) IONP@XG, (**c**) IONP@XG-NH_2_, (**d**) TC4A, and (**e**) TC4A-XG@IONP.

Fourier-transform infrared spectroscopy (FT-IR) analysis was applied to identify the functional groups of each step and the evidence for the final synthesis of TC4A-XG@IONP. The FT-IR spectra of (a) XG, (b) IONP@XG, (c) IONP@XG-NH_2_, (d) TC4A, and (e) TC4A-XG@IONP are shown in Fig. 3. The peaks about 1529, 1733, 2923, and 3386 cm^-1^ corresponded to stretching vibrations of CO–O, C=O, CH_2_, and OH of XG, shown in Fig. 3a^42^. In Fig. 3b, two peaks were added, about 575 and 1672 cm^−1^, related to C–O and Fe–O of IONP^11^. There is a characteristic peak of about 1654 cm^−1^ concerning the bending vibration of NH_2_ of IONP@XG-NH_2_ (Fig. 3c)^43^. In Fig. 3d, three characteristic peaks of TC4A were observed at 1205, 2945, and 3201 cm^−1^, which related to C–O, C–H, and O–H stretching vibrations, respectively^44,45^. Finally, in Fig. 3e, the stretch vibrations of C–O, C=C, C–H, and O–H are about 1026, 1654, 2924, and 3425 cm^−1^, which shows the complete synthesis of TC4A-XG@IONP.

#### Field emission scanning electron microscope analysis

**Figure 4:**
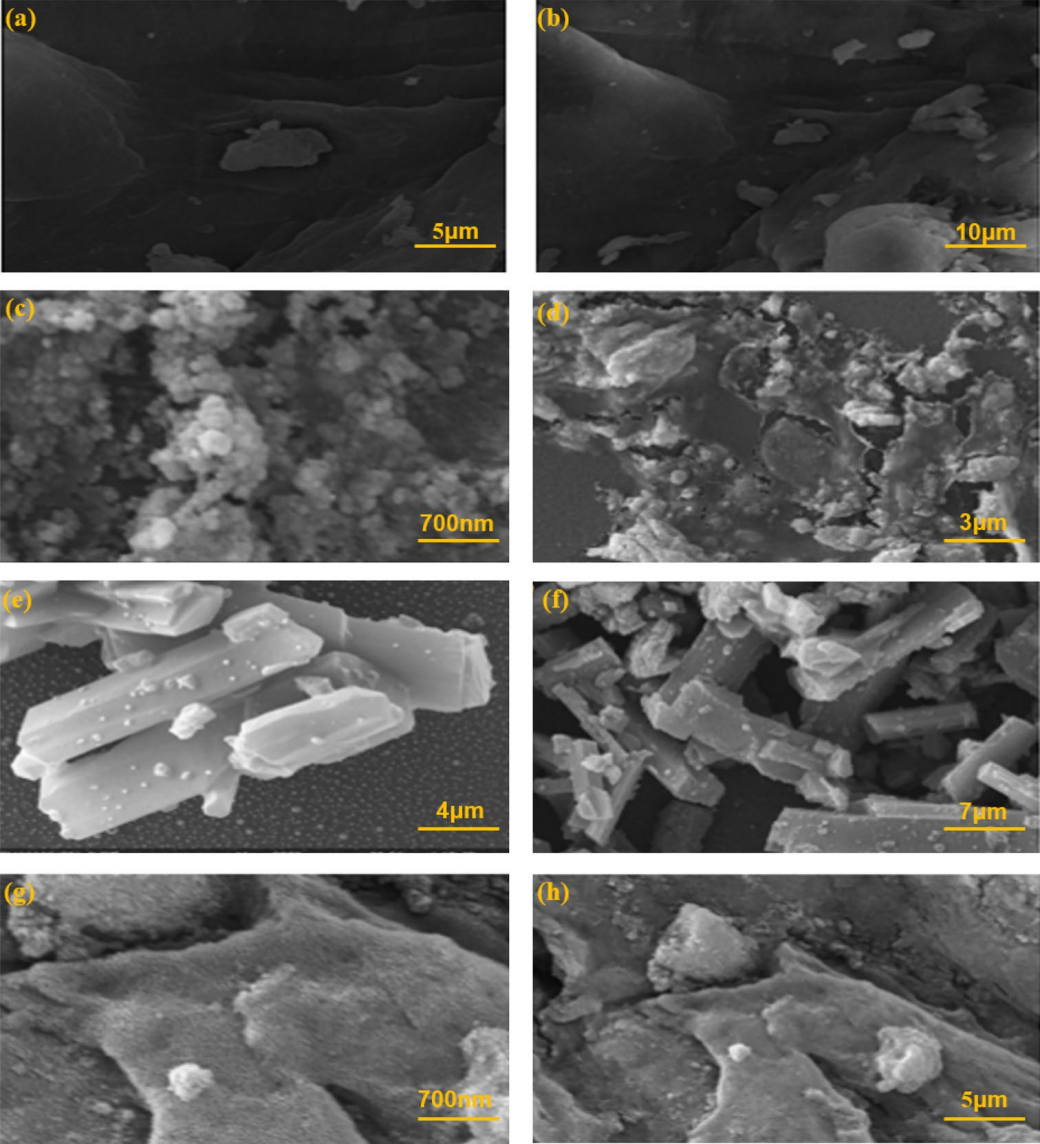
FE-SEM images of (**a** and** b**) XG, (**c** and **d**) IONP@XG, (**e** and **d**) TC4A and (**g** and **h**) the TC4A-XG@IONP.

The Field Emission Scanning Electron Microscope imaging (FE-SEM) was used to observe the particle size distribution and surface morphology of nanocatalysts XG, IONP@XG, TC4A , TC4A-XG@IONP and shown in (Fig. 4a,b) The XG image illustrate a relatively uniform matrix and non-porous structure provide a relatively smooth and regular surface, in contrast the image of IONP@XG (Fig. 4c,d) spherical nanoparticles of iron oxide are completely visible in networks without aggregation in XG polymers^46^. Also, The TC4A images (Fig. 4e,f) have an average diameter of 50 nm found in the nanoparticle structure, which consists exclusively of rod-like structures. Graph of TC4A-XG@IONP (Fig. 4g,h) exhibited the presence of TC4A on the hydrogel network of TC4A-XG@IONP by chemical attachment.

#### Thermal gravimetry analysis

**Figure 5:**
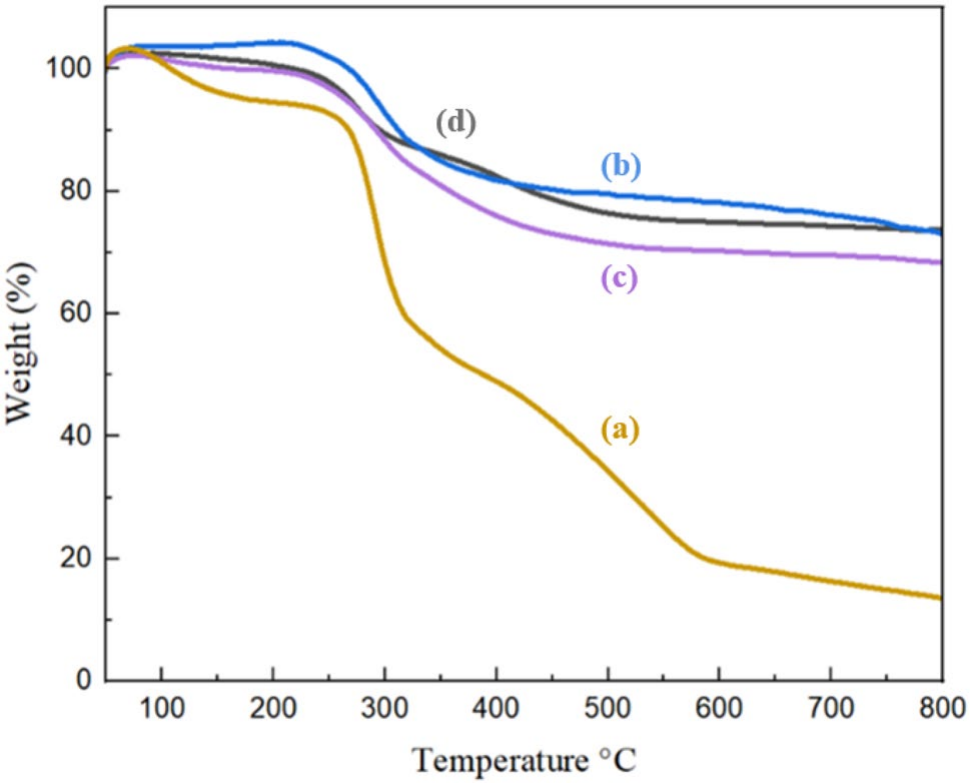
TGA Curves of (**a**) XG, (**b**) IONP@XG, (**c**) IONP@XG-NH_2_, and (**d**) TC4A-XG@IONP.

Thermal Gravimetry Analysis (TGA) evaluation of thermal resistance was performed on the samples XG, IONP@XG, IONP@XG-NH_2_, and TC4A-XG@IONP as shown in Fig. 5. TGA analysis of Fe_3_O_4_ was studied in detail to determine the thermal behavior of synthesized samples^47^. The reported thermal profile of iron oxide nanoparticles showed that its weight loss was about 5–6% up to 800 °C is related to the evaporation of surface absorbed water^9^. Figure 5a represent the thermal behavior of XG indicates that thermal degradation of this natural polymer begins at about 200 °C. The weight loss of 10% at 100 °C was due to the evaporation and outflow of water from the gum. This weight loss continued with increasing temperature, up to 300 °C, and after it went downhill^48^. About 50% of its weight is reduced between temperatures of 200–300 °C and its residuals weight at 800 °C is about 14%. The thermal profile of IONP@XG hydrogel (Illustrated in Fig. 5b) exhibited considerably elevated residual weight (73%) than unmodified XG over the studied range of temperature. This indicates the formation of iron oxide nanoparticles within the XG matrix and its effective interaction with chains of XG limits the mobility of chain of this natural polymer. Based on IONP@XG hydrogel thermogram, the first weight loss began at 270 °C which continued up to 400 °C and the second thermal decomposition observed between 400 and 800 °C. These are related to the surface dehydrogenation and dehydroxylation, dissociation of site chain and functional groups and decomposition of glycosidic bridge of XG. Thermal degradation of IONP@XG-NH_2_ as illustrated in Fig. 5c, showed similar trend with slightly decreased resudial weight (68%) as compared to IONP@XG^48^. The thermal degradation of the TC4A-XG@IONP showed three step decomposition (Fig. 5d). The initial weight loss in the temperature range of 50–200 °C caused by evaporation of adsorbed water in its cavities, the next one started at 250 °C and stained up to 300 °C which can be related to the breakdown the linkage between IONP@XG-NH_2_ and TC4A and thermal dissociation of XG functional groups. The last weight loss in the temperature range of 350–450 °C might be caused by thermal degradation of TC4A and depolymerization of XG. Modifying IONP@XG-NH_2_ with TC4A supermolecule increased its thermal resistance by 5%. Accordingly, TC4A-XG@IONP had relatively high thermal resistance with ~ 73% residual weight unto 800 °C.

#### Vibration sample magnetometer analysis

**Figure 6:**
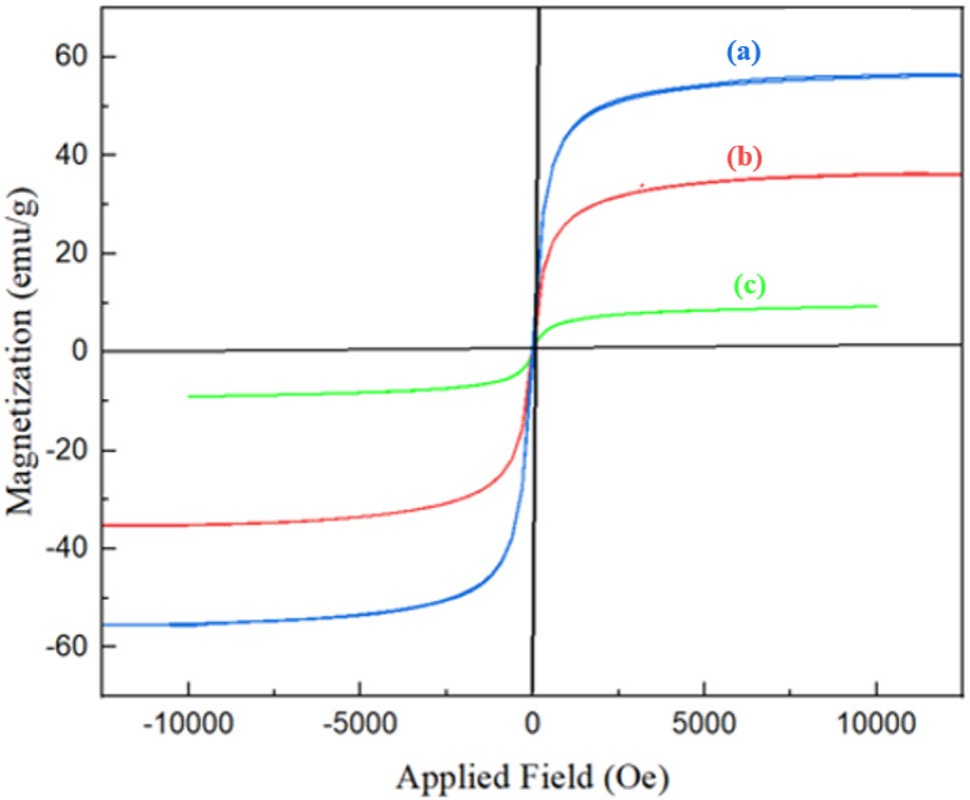
VSM analysis of (**a**) IONP, (**b**) IONP@XG, and (**c**) TC4A-XG@IONP.

The vibrating Sample Magnetometer (VSM) analysis measured the synthesized nanocatalyst magnetic properties. According to (Fig. 6a), the property magnetic coercivity and the magnetic reluctance of TC4A-XG@IONP is zero. Therefore, the synthesized samples have superparamagnetic properties. The magnetic saturation value of IONP nanoparticles is 58 emu g^−1^, and saturation magnetization of IONP@XG and TC4A-XG@IONP are decreased to 30.060 emu g^−1^ and 9.300 emu g^−1^ as illustrated in Fig. 6b,c respectively. The decrease in magnetic saturation is related to XG and TC4A, a natural polysaccharide, and a macrocyclic compound, which none of them have a magnetic nature. However, the magnetic saturation of TC4A-XG@IONP nanocatalyst is sufficient to separate it using a magnet in the experiments.

#### Brunauer–Emmett–Teller analysis

**Figure 7:**
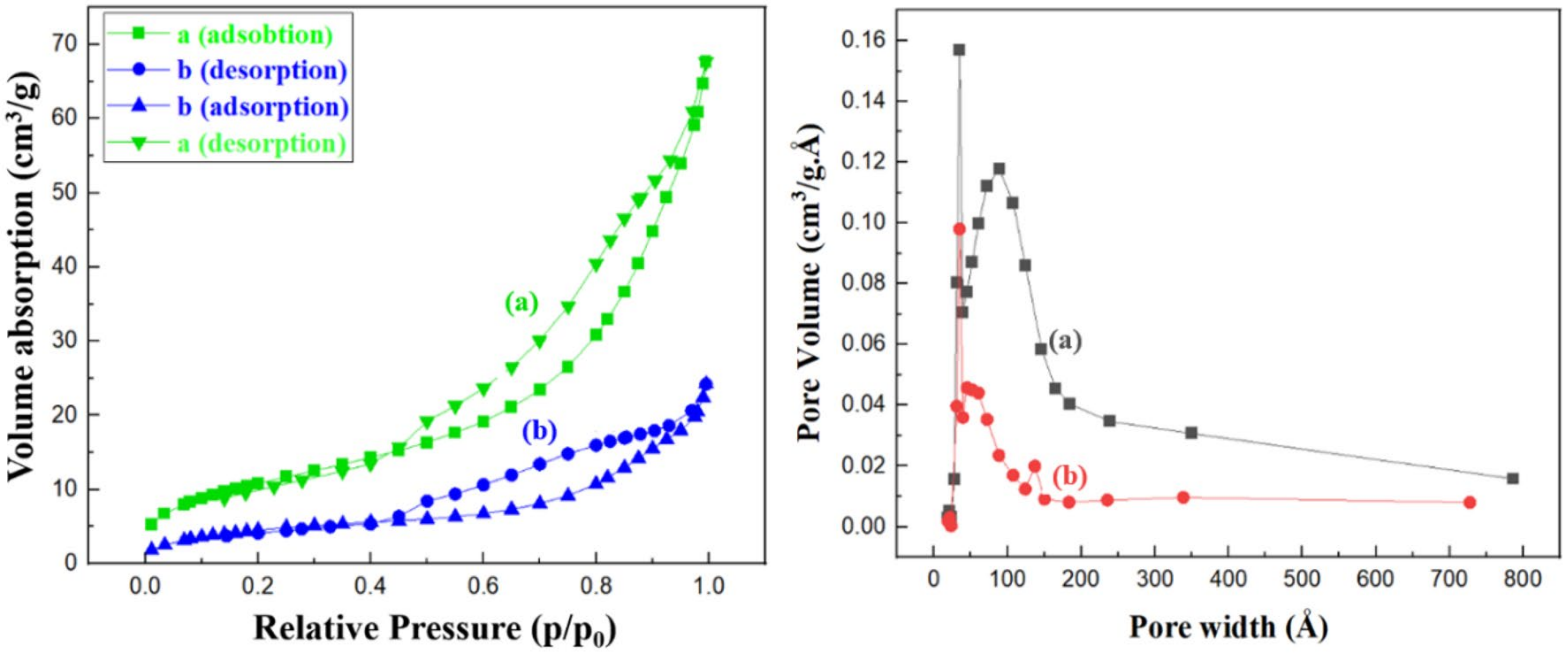
The left panel is the isotherms of N_2_ adsorption–desorption, and the right panel demonstrates the pore size distribution of (**a**) IONP@XG and (**b**) TC4A-XG@IONP.

We conducted a nitrogen adsorption–desorption analysis by Brunauer–Emmett–Teller (BET) to determine the specific surface area, porosity, and textural properties of the synthesized nanocatalyst. Based on BJH theory, Table 1 summarizes IONP@XG and TC4As-XG@Fe_3_O_4_ specific surface areas, pore volumes, and average pore diameters. Figure 7 illustrates type-IV isotherm profiles (with H4 hysteresis loops) for both nanocatalyst materials. Mesoporous materials are classified by IUPAC based on type-IV isotherm profiles. In contrast to neat XG, IONP@XG (Fig. 7a) showed a BET surface area of 45.317 m^2^ g^−1^, much higher than the 0.676 m^2^ g^−1^ previously reported for neat XG. For explanation of this observation can be said that, during the in-situ fabrication of magnetic iron oxide nanoparticles in XG natural polymer matrix, the inherent coordination potential of Fe^3+^ (trivalent metal ion) in alkaline reaction media leads to the coordination bonding between hydroxylate and carboxylate functional groups of polymeric chains of XG natural and formation of three-dimention hydrogel network with improved surface area^49^. In the case of TC4A-XG@IONP nanocatalyst (Fig. 7b), the BET surface area was measured 17.3065 m^2^ g^−1^, which was satisfactory compared to IONP@XG. The surface area and size of TC4A-XG@IONP were decreased following, amine-functionalization, and then covalent bonding to funtionalized TC4A. A nanocatalyst fabricated with favorable textural properties, a porous structure, and a high specific surface area may be considered as a desired catalytic system.

**Table 1:** BET Surface area, pore volume and pore size of IONP@XG and TC4A-XG@IONP nanocatalyst.

Sample	Surface area^a^ (m^2^.g^-1^)	Pore volume^b^ (cm^3^.g^-1^)	Pore size^b^ (nm)
IONP@XG	45.317	0.106	9.377
TC4A-XG@IONP	17.306	0.038	9.309

^a^The surface area parameter was acquired via BET analysis.

^b^The pore volume and pore size parameters were acquired via BJH analysis.

#### X-Ray diffraction analysis

**Figure 8:**
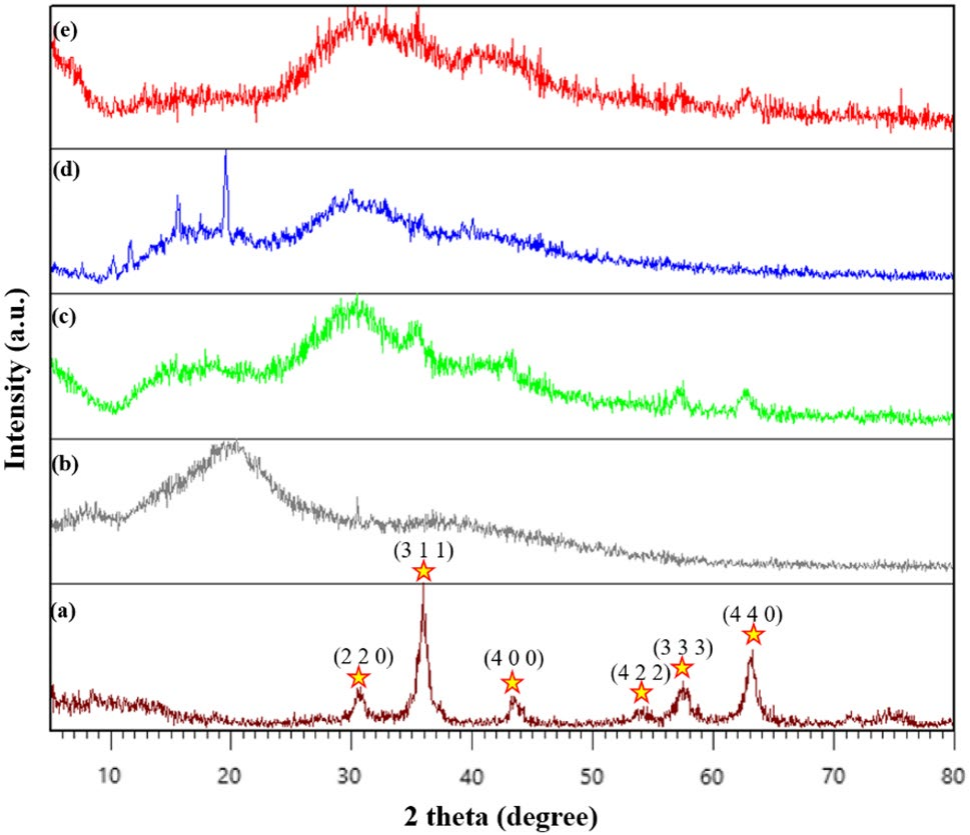
XRD patterns (**a**) of IONP, (**b**) XG, (**c**) IONP@XG, (**d**) TC4A, and (**e**) TC4A-XG@IONP.

The crystallinity of the produced materials in the range of 5–80° was investigated using X-Ray diffraction analysis (XRD) analysis^50^. According to earlier research, a distinctive peak (at 2θ = 18°–25°) was seen in the XRD pattern of XG, indicating that it is an amorphous material (Fig. 8b). As shown in Fig. 8a, the acceptable IONP pattern with card number JCPDS, 01-77-0010 fits the XRD pattern for LONP, which includes diffraction peaks at 2θ = 30.61° 35.99°, 43.27°, 54.18°, 57.53° and 63.35°. The same peaks as those associated with IONP have been identified for IONP@XG, but since XG is an amorphous material, their pitch is less intense (Fig. 8c). According to studies in the literature, TC4A has a crystalline structure in Fig. 8d. Additionally, the typical IONP peak can be seen in the XRD pattern of the TC4A-XG@IONP nanocatalyst, confirming the existence of IONP MNPs there. Finally, it can be said that compared to pristine XG, *in-situ* magnetization,amine modification and functionalization with TC4As produced better crystallinity(Fig. 8e).

#### Energy dispersive X-ray

**Figure 9:**
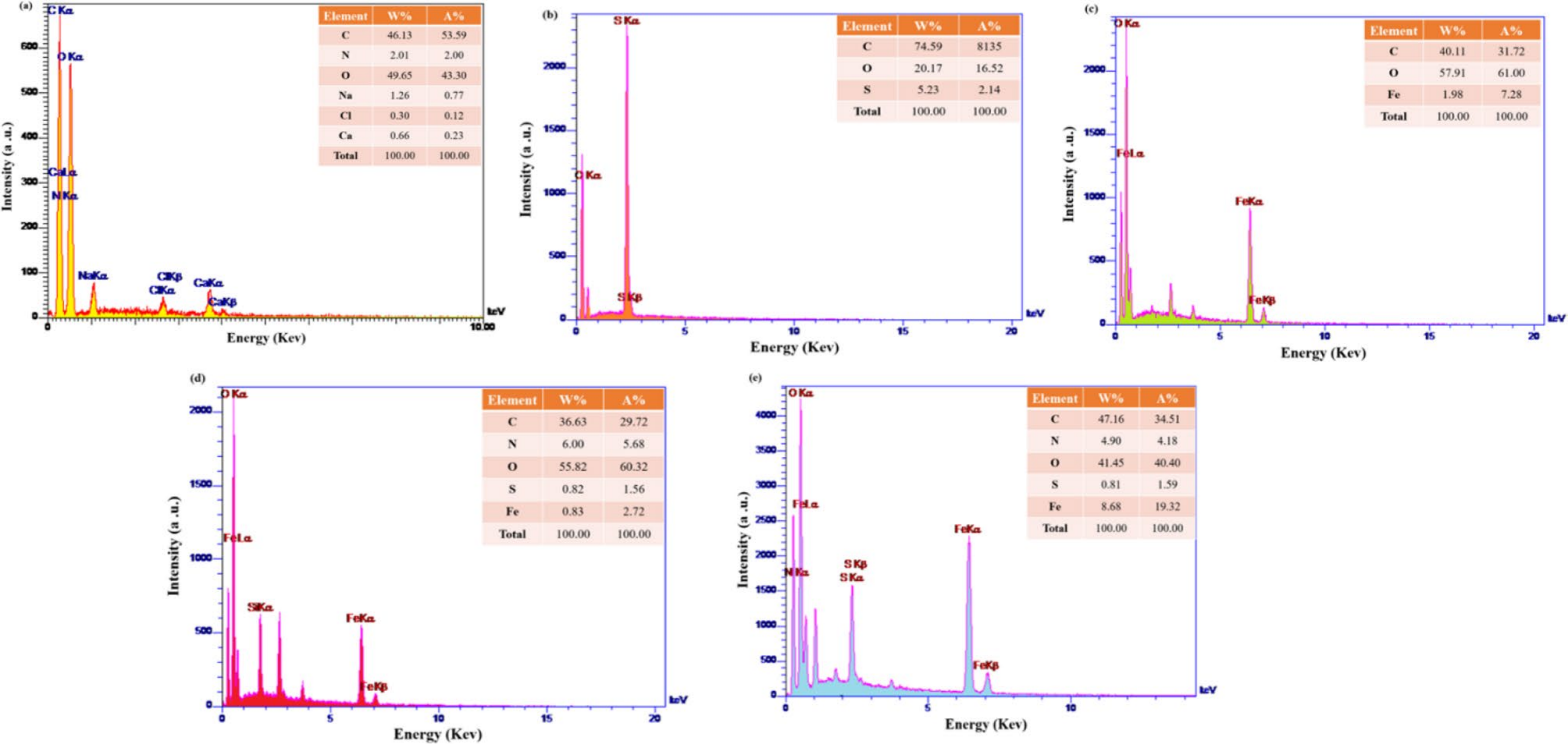
EDX patterns (a) of (**a**) XG, (**b**) TC4A, (**c**) IONP@XG, (**d**) IONP@XG-NH_2_, and (**e**) TC4A-XG@IONP.

The detection and identification of organic and inorganic elements in the manufactured compounds was done by the Energy Dispersive X-Ray (EDX) qualitative analysis method^51,52^. Therefore, the constituent elements of the prepared samples can be examined in the EDX spectrum. As shown in Fig. 9a–e, the EDX spectrum of neat XG was illustrated in Fig. 9a with high intensity peaks represent to C, N, O, Na, Cl and Ca elements. On the analysis of the TC4A, the weight% (atomic%) values for C, O, and S are 74.59% (81.35%), 20.17% (16.52%), and 5.23% (2.14%) respectively,which are the main constituents of TC4A, are well visible in Fig. 9b. In the spectrum Fig. 9c the elements C, O and Fe are confirm the structure of IONP@XG. Spectrum Fig. 9d confirm the structure of IONP@XG-NH_2_ with the relayted elements (C, O, N, Si, and Fe). The peaks related to the final nanocatalyst (TC4A-XG@IONP), this diagrame also has high intensity peaks of the elements including C, O, N, S, and Fe are the main constituent elements that are shown with high intensity (Fig. 9e). A uniform distribution of elements is observed in (a) XG, (b) TC4A, (c) IONP@XG, (d) IONP@XG-NH_2_, and (e) TC4A-XG@IONP, as illustrated in Fig. S6 of the Supplementary Materials.

### Catalytic study

The green and eco-friendly TC4A-XG@IONP was applied as a catalyst in organic reaction. For this purpose, it was used as a catalyst in synthesizing acridinedione derivatives. The one-pot reaction between dimedone, 4-chlorobenzaldehyde, ammonium acetate/aniline/substituted aniline, and ethanol as solvent was carried out as a model reaction. Different conditions like temperature, amount of catalyst, solvent, and reaction time were investigated and shown in Table 2. First, the reaction was performed without a catalyst at room temperature (rt) and 80 °C and observed there was no product, which shows the essential role of the catalyst (Table 2, Entry 1 and 2). Then, the reaction was applied at rt, 40 °C, and 80 °C, it was observed at 80 °C, the product yield was the highest (Table 2, Entry 3–5). Other green solvents including water, methanol, and acetonitrile were investigated, and ethanol as a solvent has the highest yield (Table 2, Entry 6–8). Also, the amount of catalyst was investigated, which observed that 20 mg catalyst is the optimized amount, and the extra amount does not affect yield (Table 2, Entry 9 and 10). The reaction time was also investigated at 10, 20, and 30 min. It was found that the optimized reaction time is 20 min, and more than, does not affect the yield of the intended product (Table 2, Entry 11–13). Moreover, the reaction performed by XG and TC4A. The results showed that the TC4A-XG@IONP nanocatalyst has more yield, which shows the merits of its catalytic activity (Table 2, Entry 14 and 15).

**Table 2:** Different reaction conditions of synthesizing acridinedione derivatives^a,b^.

Entry	Catalyst	Amount (g)	Temperature (°C)	Time (minutes)	Solvent	Yield (%)
1	–	0.01	rt	10	EtOH	–
2	–	0.01	80	20	EtOH	–
3	TC4A-XG@IONP	0.01	rt	20	EtOH	–
4	TC4A-XG@IONP	0.01	40	20	EtOH	68
5	TC4A-XG@IONP	0.01	80	20	EtOH	92
6	TC4A-XG@IONP	0.01	80	20	water	76
7	TC4A-XG@IONP	0.01	80	20	MeOH	62
8	TC4A-XG@IONP	0.01	80	20	Acetonitrile	48
9	TC4A-XG@IONP	0.01	80	20	EtOH	92
10	TC4A-XG@IONP	0.05	80	20	EtOH	92
11	TC4A-XG@IONP	0.01	80	10	EtOH	55
12	**TC4A-XG@IONP**	**0.01**	**80**	**20**	**EtOH**	**92**
13	TC4A-XG@IONP	0.01	80	30	EtOH	92
14	XG@IONP	0.01	80	10	EtOH	79
15	XG-TC4A	0.01	80	10	EtOH	76
16	IONP	0.01	80	10	EtOH	69
17	TC4A	0.01	80	10	EtOH	63

^a^Reaction conditions: 4-chlorobenzaldehyde (1 mmol), hydrazine hydrate (1 mmol), ethyl acetoacetate (1 mmol), malononitrile (1 mmol) catalyst (0.02 g), and ethanol (5 mL) refluxed in 80 °C.

^b^Yields refer to pure products.

Significant values are in bold.

After obtaining the optimized reaction conditions, various aldehyde and amine derivatives were used to show the TC4A-XG@IONP nanocatalytic merits, and the intended products were obtained with high yields (Table 3).

**Table 3:** Synthesis of acridinedione derivatives by TC4A-XG@IONP^a,b^.


Entry	R	Amine	Product	Mp (°C, [Ref])	Yield (%)
A	–H	NH_4_OAc	5a	280–282^53^	90
B	–2,4 di OMe	NH_4_OAc	5b	244–245^54^	82
C	–4 Cl	NH_4_OAc	5c	302–304^55^	92
D	–4 OH	NH_4_OAc	5d	301–303^56^	85
E	–3 NO_2_	NH_4_OAc	5e	294–296^57^	87
F	–4 NO_2_	NH_4_OAc	5f.	299–300^58^	89
G	–4 CN	NH_4_OAc	5 g	271–273^59^	88
H	–4 Br	NH_4_OAc	5 h	236–237^60^	85
I	–4 OH	Aniline	6a	251–252^61^	83
J	–4 Cl	Aniline	6b	241–243^62^	81
K	–4 NO_2_	Aniline	6c	291–292^63^	85
L	–4 Cl	4-OH Aniline	6d	332–334^64^	72
M	–4 Cl	4- Cl Aniline	6e	302–304^65^	81
N	–4 Cl	3- NO_2_ Aniline	6f.	280–283^66^	74
O	–4 Cl	2-Me-Aniline (o-Toluidine)	6 g	203–204 (this work)	88

^a^Reaction conditions: aromatic aldehyde (1 mmol), hydrazine hydrate (1 mmol), ethyl acetoacetate (1 mmol), malononitrile (1 mmol) catalyst (0.02 g), and ethanol (5 mL) refluxed in 80 °C.

^b^Yields refer to pure products.

In addition, the catalytic activity of TC4A-XG@IONP nanocatalyst comparisons was made with some other catalysts reported (Table 4) and observed that using the synthesized nanocatalyst has a higher yield than the others. Based on this, it can be concluded that TC4A-XG@IONP nanocatalyst is an efficient catalyst in synthesizing acridinedione derivatives.

**Table 4:** Comparing catalytic activity of TCA_XG@IONP nanocatalyst with other reported catalysts.

Entry	Catalyst	Reaction Conditions	Yield (%)	Refs.
1	MFRH	Solvent free-100 °C	80	^67^
2	MOFs	Solvent free- 125 °C	78	^19^
3	Nano ferrite	Toluene -130 °C	77	^68^
4	HY-Zeolite	EtOH-Reflux	77	^69^
5	TC4A-XG@IONP	EtOH-Reflux	92	This work

### Proposed mechanism

The green and multifunctional TC4A-XG@IONP nanocatalyst can perfectly accelerate organic reactions due to the desired physicochemical properties as follows; large surface area, high porosity, three-dimentional network, and abundantly OH and NH_2_ groups. The plausible mechanism for synthesizing acridinedione derivatives with TC4A-XG@IONP as a nanocatalyst. First, the catalyst makes a hydrogen bonding with dimedone and makes the alpha hydrogen highly acidic. Then, aromatic aldehyde was activated by TC4A-XG@IONP nanocatalyst, and dimedone made a nucleophilic attack and a knovenagel product was obtained. By dehydration, a Michael reaction would occur with another dimedone with acidic hydrogen through hydrogen bonding with TC4A-XG@IONP nanocatalyst. Then ammonium acetate would react with the carbonyl group, and the intended product would be obtained with the help of a nanocatalyst by intramolecular cyclization and dehydration (Fig. S5).

### Reusability

**Figure 10:**
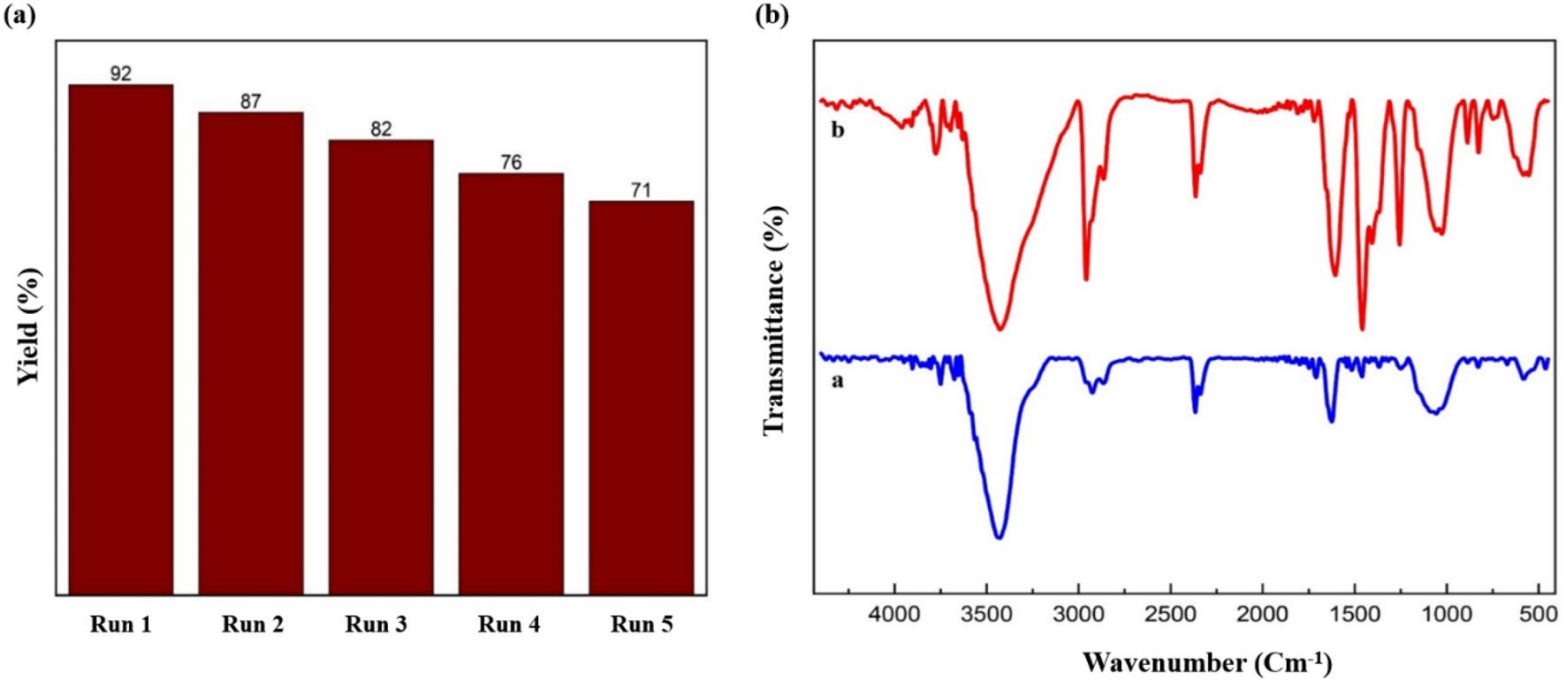
(**a**) Recycling diagram of TC4A-XG@IONP nanocatalyst in synthesizing of the acridinedione derivative (5c) and (**b**) FT-IR of (**a**) TC4A-XG@IONP nanocatalyst and (**b**) recycled TC4A-XG@IONP nanocatalyst.

In green chemistry, the ability to reuse the catalyst is one of the main principles. So, the reusability of TC4A-XG@IONP nanocatalyst was investigated in synthesizing the acridinedione derivatives. This process involved extracting the TC4A-XG@IONP nanocatalyst from a mixture with an external magnet, washing it several times with ethanol and water, drying it in an oven, and reusing it for the reaction. As shown in Fig. 10a, the TC4A-XG@IONP nanocatalyst was reused four times with no apparent diminish in the product yield. Also, the FT-IR of reused TC4A-XG@IONP is shown in Fig. 10b, and it is clear that the structure maintained its stability.

## Conclusion

In the present work, we constructed a composition of XG and TC4A via covalent bonding in the presence of IONP and by using organosilane, and epichlorohydrine. The next step, the employed characterization methods have confirmed well the construction of TC4A-XG@IONP nanocatalyst. We then used that nanocatalyst to synthesize the acridinedione derivatives through one-pot multicomponent reaction of dimedone, various aromatic aldehydes, and different amines (ammonium acetate or aniline/substituted aniline). The corresponding products were synthesized with good to high yields (92–72%). The presented eco-friendly catalytic system has great textural and structural characteristics which originated from its constituent components, (i.e., XG, IONP, and TC4A) and their synergistic effects such as high porosity and presence of cavity shaped structure, high surface area, abundant reactive functional groups as catalytic sites, high thermal stability, and great retrievability, considering the great advantages that this nanocatalyst has for the acridinedione derivatives, still has challenges such as scalability challenge researchers could explore innovative approaches for producing the nanocatalyst on a larger scale while maintaining its catalytic activity and stability. This could involve investigating alternative synthesis methods or optimizing the current synthesis process to reduce production costs and increase yield. To evaluate the potential environmental impact of the nanocatalyst, researchers could conduct comprehensive toxicity studies to assess its effects on human health and the environment. They could also investigate methods for safely disposing of or recycling the nanocatalyst after use to minimize any negative environmental impacts.Overall, addressing these challenges will be crucial for advancing the development and application of the TC4A_XG@IONP nanocatalyst as a sustainable and efficient alternative for organic transformations in various industrial sectors.

### Supplementary Information

Supplementary Figures.

## Data Availability

The datasets used and/or analyzed during the current study available from the corresponding author on reasonable request.
