# Supplementary material for: Utilizing magnetic xanthan gum nanocatalyst for the synthesis of acridindion derivatives via functionalized macrocycle Thiacalix[4]arene
Source: Sci Rep. 2023 Dec 13;13:22162. doi: 10.1038/s41598-023-49632-x (PMC10719371; doi:10.1038/s41598-023-49632-x)
Supplement: Supplementary file 1 — Supplementary Figures. [file 41598_2023_49632_MOESM1_ESM.docx]

| Supporting information |
| --- |

**Utilizing Magnetic Xanthan Gum Nanocatalyst for the Synthesis of Acridindion Derivatives via Functionalized Macrocycle Thiacalix [4]arene**

*Fereshte Hassanzadeh-Afruzi^†^, Mohammad Mehdi Salehi^†^, Ghazaleh Ranjbar, Farhad Esmailzadeh, Peyman Hanifehnejad*, *Mojtaba Azizi, Faten Eshrati yeganeh,* Ali Maleki^*^

*Catalysts and Organic Synthesis Research, Laboratory, Department of Chemistry, Iran University of Science and Technology, Tehran, Iran*

**Corresponding authors: (A. Maleki) E-mail:* [*maleki@iust.ac.ir*](mailto:maleki@iust.ac.ir)*; Fax: +98-21-73021584; Tel: +98-21-73228313.*

*† These authors contributed equally to this work.*


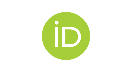
*Author’s ORCIDs:*

*Fereshte Hassanzadeh-Afruzi:*[*https://orcid.org/0000-0003-0570-2506*](https://orcid.org/0000-0003-0570-2506)

*Mohammad Mehdi Salehi: https://orcid.org/0000-0003-3648-1865*

*Farhad Esmailzadeh :https://orcid.org/0000-0003-4551-8180*

*Ali Maleki: https://orcid.org/ 0000-0001-5490-3350*

| **Content** | **Page** |
| --- | --- |
| **Figure S1:** FTIR Spectrum of 6g | **S2** |
| **Figure S2:** ^1^H NMR Spectrum of 6g (expanded) | **S3** |
| **Figure S3:** FTIR Spectrum of 5c | **S4** |
| **Figure S4.** ^1^H NMR Spectrum of 5c (expanded) | **S5** |
| **Figure S5.** Schematic of proposed mechanism | **S6** |
| **Figure S6.** Elements distribution | **S7** |

**Figure S1:** FTIR Spectra of 9-(4-chlorophenyl)-3,3,6,6-tetramethyl-10-(o-tolyl)-3,4,6,7,9,10 hexahydroacridine-1,8(2H,5H)-dione (6g)


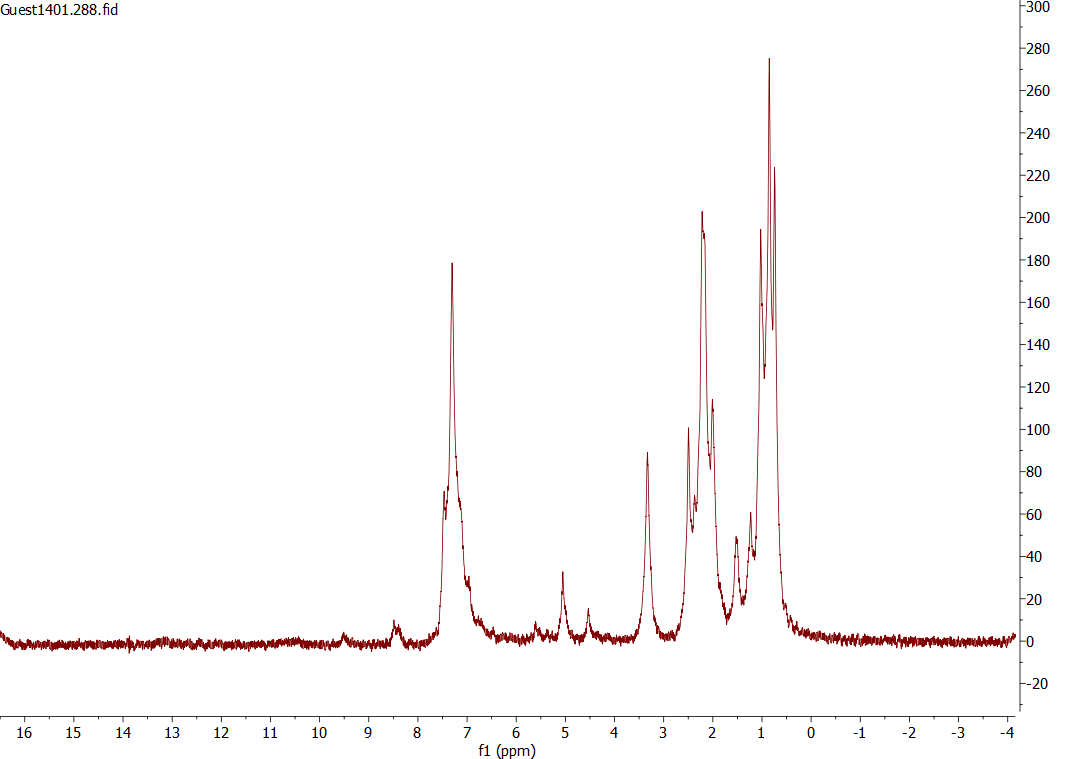


**Figure S2:** ^1^H NMR (500 MHz, CDCl_3_) 9-(4-chlorophenyl)-3,3,6,6-tetramethyl-10-(o-tolyl)-3,4,6,7,9,10-hexahydroacridine-1,8(2H,5H)-dione (6g).


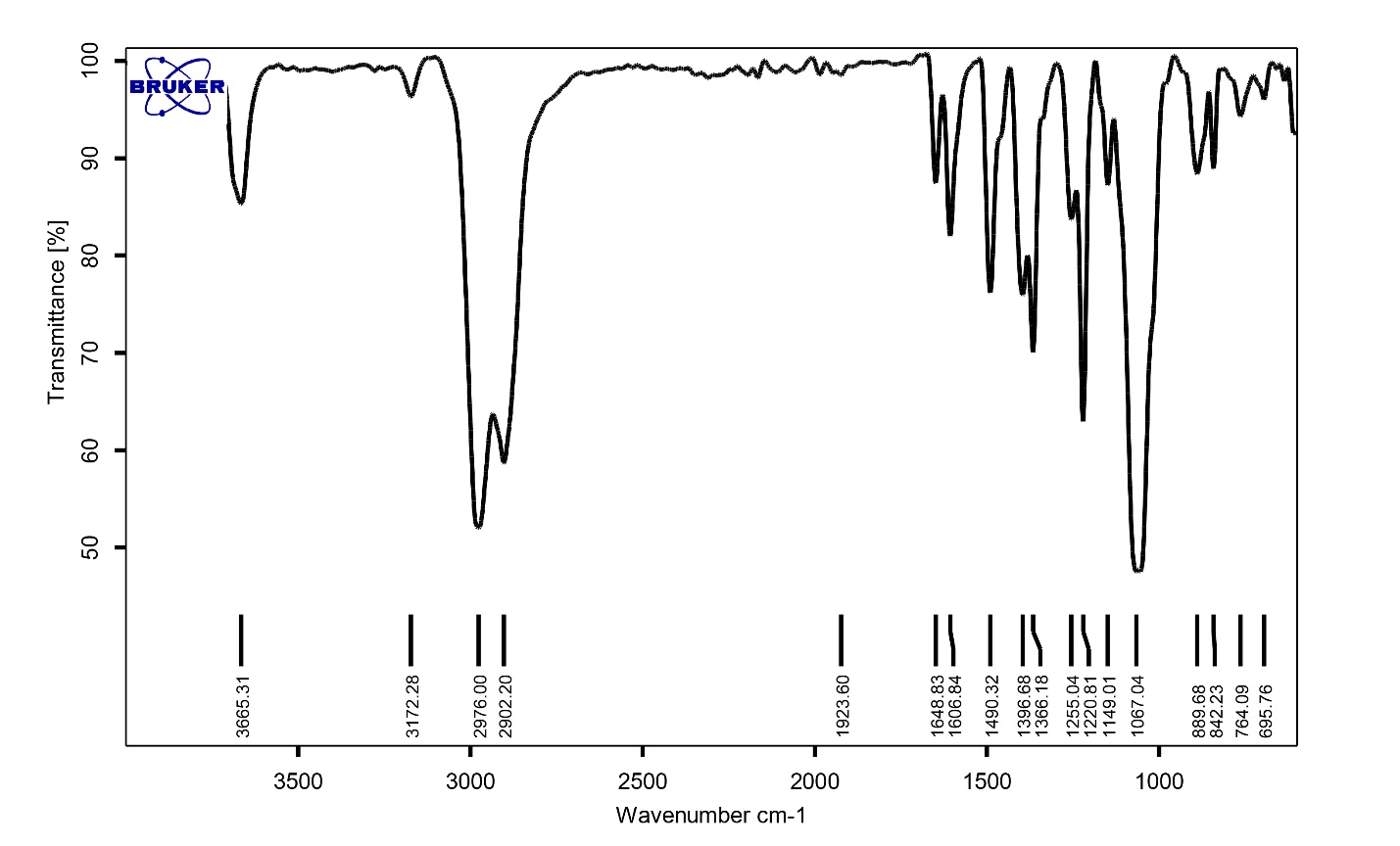

**Figure S3:** 3,3,6,6-Tetramethyl-9-(4-chlorophenyl)-3,4,6,7,9,10-hexahydroacridine 1,8(2H,5H)-dione (5c).


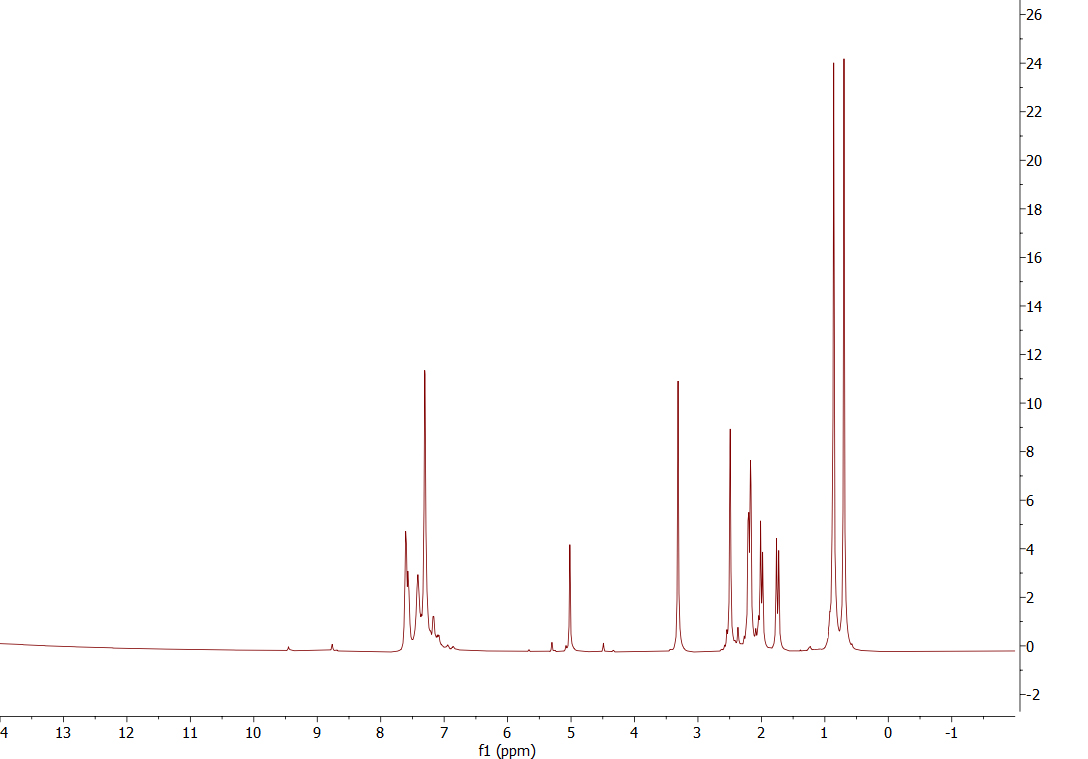

**Figure S4:** ^1^H NMR (500 MHz, CDCl_3_) 3,3,6,6-Tetramethyl-9-(4-chlorophenyl)-3,4,6,7,9,10-hexahydroacridine 1,8(2H,5H)-dione (5c).

**
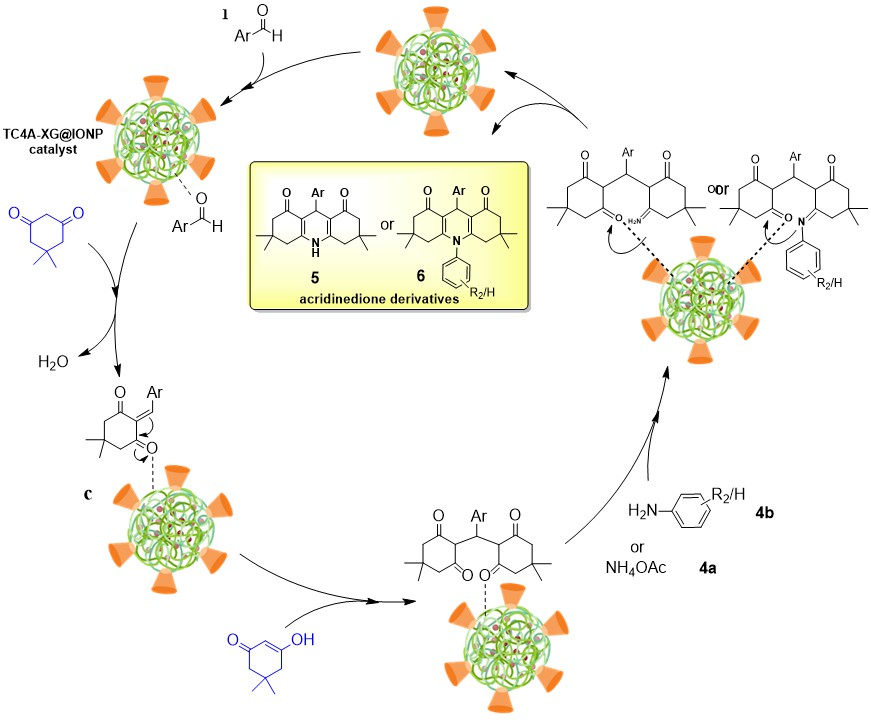
**

**Figure S5:** A plausible mechanism for catalyzing the synthesis reaction of acridinedione derivativesby TC4A-XG@IONP

**
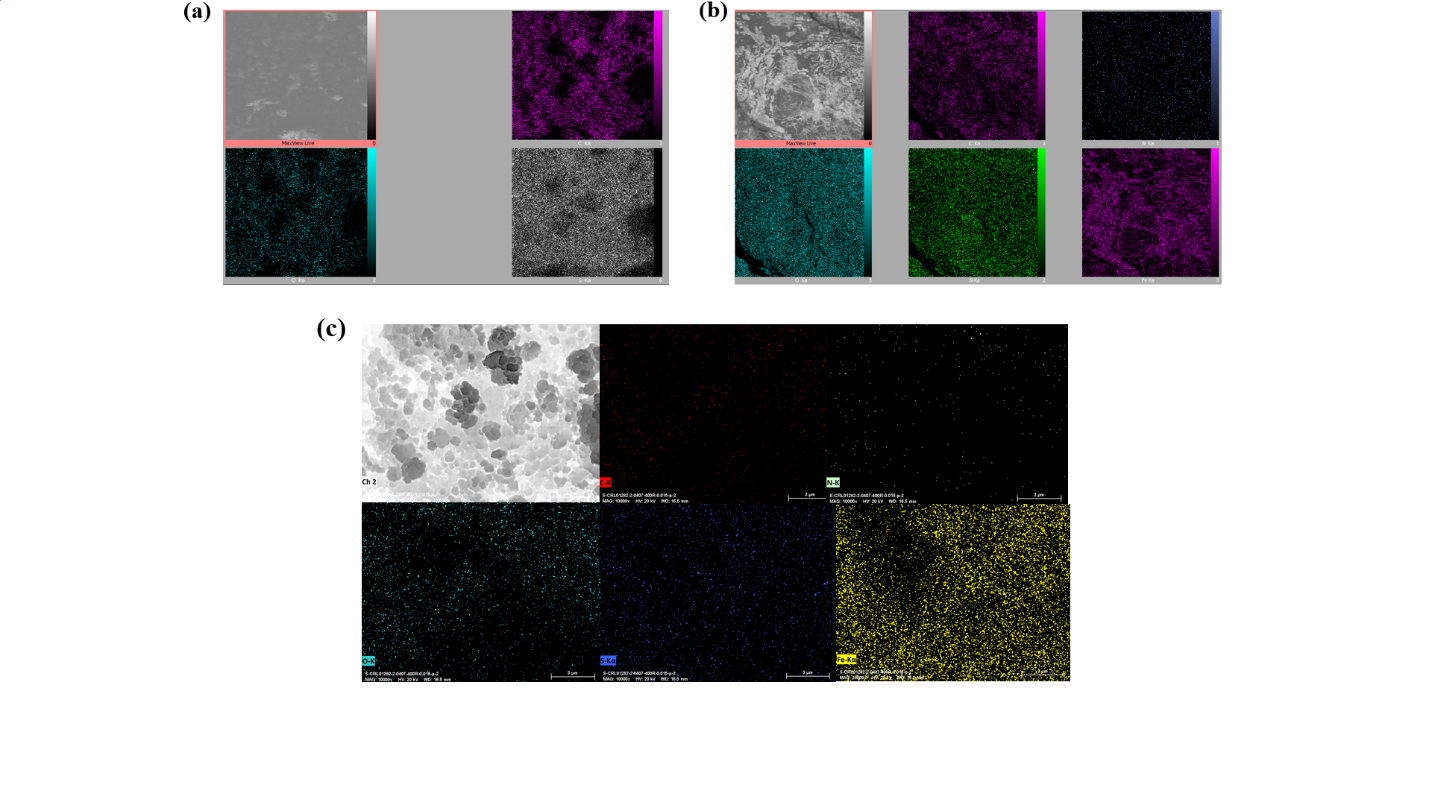
**

**Figure S6:** Elements distribution (EDX mapping) a) TC4A, b) IONP@XG_NH_2_, and c) TC4A-XG@IONP.
